# Paracrine Shear-Stress-Dependent Signaling from Endothelial Cells Affects Downstream Endothelial Function and Inflammation

**DOI:** 10.3390/ijms222413300

**Published:** 2021-12-10

**Authors:** Fabio Bertani, Dalila Di Francesco, Maria Dolores Corrado, Maria Talmon, Luigia Grazia Fresu, Francesca Boccafoschi

**Affiliations:** 1 Laboratory of Human Anatomy, Department of Health Sciences, University of Piemonte Orientale, 28100 Novara, Italy; fabio.bertani@studenti.uniupo.it (F.B.); dalila.di-francesco.1@ulaval.ca (D.D.F.); 20019628@studenti.uniupo.it (M.D.C.); 2 Laboratory of Pharmacology, Deaprtment of Health Sciences, University of Piemonte Orientale, 28100 Novara, Italy; maria.talmon@med.uniupo.it (M.T.); luigia.fresu@med.uniupo.it (L.G.F.)

**Keywords:** endothelial dysfunction, microvascular dysfunction, shear stress, mechanotransduction, inflammatory response in endothelial cells

## Abstract

Cardiovascular diseases (CVDs), mainly ischemic heart disease (IHD) and stroke, are the leading cause of global mortality and major contributors to disability worldwide. Despite their heterogeneity, almost all CVDs share a common feature: the endothelial dysfunction. This is defined as a loss of functionality in terms of anti-inflammatory, anti-thrombotic and vasodilatory abilities of endothelial cells (ECs). Endothelial function is greatly ensured by the mechanotransduction of shear forces, namely, endothelial wall shear stress (WSS). Low WSS is associated with endothelial dysfunction, representing the primary cause of atherosclerotic plaque formation and an important factor in plaque progression and remodeling. In this work, the role of factors released by ECs subjected to different magnitudes of shear stress driving the functionality of downstream endothelium has been evaluated. By means of a microfluidic system, HUVEC monolayers have been subjected to shear stress and the conditioned media collected to be used for the subsequent static culture. The results demonstrate that conditioned media retrieved from low shear stress experimental conditions (LSS-CM) induce the downregulation of endothelial nitric oxide synthase (eNOS) expression while upregulating peripheral blood mononuclear cell (PBMC) adhesion by means of higher levels of adhesion molecules such as E-selectin and ICAM-1. Moreover, LSS-CM demonstrated a significant angiogenic ability comparable to the inflammatory control media (TNFα-CM); thus, it is likely related to tissue suffering. We can therefore suggest that ECs stimulated at low shear stress (LSS) magnitudes are possibly involved in the paracrine induction of peripheral endothelial dysfunction, opening interesting insights into the pathogenetic mechanisms of coronary microvascular dysfunction.

## 1. Introduction

Initially described in 1865 by the Swiss anatomist Wilhelm His [1,2] as a mere inert barrier separating blood from the surrounding tissues, from the 1970s the endothelium has been increasingly associated with vascular performance. In fact, it has an essential role in maintaining blood fluidity, driving platelet aggregation and vascular tone; moreover, it is a major actor in the regulation of immune response, inflammation, angiogenesis, and, lastly, it is also a metabolizing and endocrine organ [3]. Due to the luminal localization, endothelial cells (ECs) are masters in sensing hemodynamic and mechanical changes; therefore, they respond accordingly [4].

One of the most important mechanical stimuli for endothelial cells is called wall shear stress (WSS), which is the frictional force exerted by the blood flow applied tangentially to the endothelium, expressed as force/unit area (N/m^2^ or Pascal or dynes/cm^2^). WSS is sensed by ECs by means of a wide variety of cellular structures and organelles such as endothelila cilia, glycocalyx and caveolae [5]. One of the most important pathways is the mechano-sensory complex that localizes at cell–cell junctions [6,7,8]. This multi-component complex consists of platelet endothelial cell adhesion molecule 1 (PECAM-1), vascular endohelial-cadherin and vascular endothelial growth factor receptor 2 (VEGFR-2), which have been indicated to be responsible for the activation of shear-response pathways ranging from cell alignment to endothelial function and dysfunction [6,9,10,11].

(1) $$\tau =\eta \frac{\partial U}{\partial y}{|}_{y=0}$$

Mathematically, wall shear stress can be determined as in Equation (1), where *ƞ* is the fluid viscosity, while $\frac{\partial U}{\partial y}$ is the partial derivative of the velocity with respect to the spatial coordinate *y* (i.e., the velocity gradient) when *y* is equal to 0 (i.e., the vessel boundary). Wall shear stress is, thus, strictly related to fluid viscosity, which can be conceptualized as the frictional force preventing the relative movement of the adjacent layers of fluid. 

Phenotypically, WSS determines changes in cell morphology resulting in cytoskeletal reorganization and alignment [6], but the most significant change is an increased nitric oxide (NO) release, which directly correlates with WSS increase in magnitude [12]. NO is produced from L-arginine by a specific enzyme, called endothelial nitric oxide synthase (eNOS), located in membrane caveolae or on Golgi apparatus. When cells are exposed to physiological or supra-physiological shears, this enzyme is promptly upregulated both at protein and gene regulatory levels [13,14]. Within seconds after shear stress stimulation, eNOS is activated showing an increase in NO, while over several hours shear stress stimulates an increase in eNOS mRNA and protein expression [14]. This occurs because of multiple cis-regulatory DNA sequences, such as SP-1, GATA, activator protein-1, activator protein-2, nuclear factor-1, shear stress response elements (SSRE) and sterol-regulatory elements (SRE) [15].

Hence, unaltered eNOS activity is a pivotal goal that endothelial cells must achieve to maintain vascular homeostasis. Several observations indicated that high shear stress (i.e., 10–30 dyn/cm^2^ for veins and 15–100 dyn/cm^2^ for arteries) [16], typically present in straight segments of blood vessels, protects against atherogenic stimuli [9]. By contrast, lower values of flow patterns that involve changes in the direction and magnitude of flow, induce a proinflammatory and prothrombotic state characterized by progressive endothelial dysfunction [17]. The molecular mechanisms correlating low shear stress stimulation and increased proinflammatory phenotype remain largely unexplored, even though recent findings revealed that low WSS is associated with increased O_2_·^−^ and phosphorylation on Thr495, markers of eNOS uncoupling [18,19]. According to these findings, reduced expression and activity of eNOS is considered to be the *primum movens* in endothelial dysfunction. Low shear stress regions are physiologically found throughout the vascular tree. In particular, inner curvatures, branches and bifurcations are characterized by focal patches in which blood flow is decelerated, oscillatory or turbulent [20,21]. These regions are considered to be at high risk of losing endothelial function and therefore promoting atherosclerotic plaque initiation, as reported by several in vivo studies [22,23,24,25,26,27,28] and epidemiological studies [29,30,31,32], deepening the correlation between plaque localization and shear stress. In humans, the tendency to develop atheroma plaques in the external side of the vessel curvature of carotid arteries at the level of the bifurcation of the common carotid artery, is indeed well documented [31,33]. 

In the coronary arteries, the preferential sites for plaque initiation and progression are in segments with bifurcations as well as along curvatures’ inner walls [34,35,36]. These athero-susceptible regions typically show increased permeability to plasma molecules, due to leaky junctions between ECs, increased cellular turnover and increased adhesiveness of monocytes [37]. Hence, together with traditional risk factors such as smoking, hyperlipidemia, hypertension, metabolic syndrome or diabetes, endothelial WSS plays a significant role in focal atherosclerotic plaque formation and development, together with plaque vulnerability and adverse cardiac prognosis [38,39,40,41]. In 2018, Siasos and colleagues performed a retrospective cohort study trying to elucidate the correlation between epicardial endothelial dysfunction (considered as vasodilation/vasoconstriction) and microvascular endothelial dysfunction (defined as an increase in coronary flow), in patients with mild coronary atherosclerosis [38]. The researchers found that epicardial coronaries, upstream to regions with abnormal microvascular function, exhibited lower WSS compared with arteries upstream to functional microvasculature, suggesting a significant association between coronary microvascular dysfunction and endothelial dysfunction in non-obstructive coronary artery disease (CAD) patients. Interestingly, this research also found that microvascular dysfunction temporally preceded epicardial coronary dysfunction, pointing to hemodynamic factors as a possible cause in the periphery destabilizing the up-stream local WSS [42]. Despite the precise insight into the correlation between microvascular and epicardial dysfunction, it remains quite difficult to draw a causal relationship between these two phenomena. 

In order to give additional experimental evidence on endothelial cell functionality and altered shear stress, the present work attempted to evaluate the possible peripheral effect of regions proximally exposed to low shear stress magnitudes. Endothelial cells will be subjected to different magnitudes of shear stress for 24 h and the conditioned media will be collected and used for the static culture of other monolayers of endothelial cells, therefore simulating the peripheral effect exerted by factors released by differently sheared endothelia.

Here, we demonstrated that endothelial cells challenged at different shear stress values release different factors affecting, in a paracrine way, downstream endothelial cells. These factors are responsible for the modulation of endothelial function, inflammation and finally vascular remodeling and angiogenesis. 

## 2. Results

### 2.1. Shear-Stress-Challenged HUVEC

HUVECs were seeded in two different PPFCs. Y-shaped PPFCs were used for fluorescence experiments allowing for the contemporary evaluation of two shear stress conditions within the same experiment. I-Luer PPFCs were used in all the other experiments requiring cell lysis for the analysis. This kind of microfluidic chambers were also used for the experiments requiring the production and collection of different conditioned media (CMs). HUVECs were challenged, in both experimental designs at 6 dyn/cm^2^ (i.e., low shear stress—LSS) and 10 dyn/cm^2^ (i.e., high shear stress—HSS).

#### 2.1.1. Shear Stress Induces the Alignment of HUVECs

**Figure 1:**
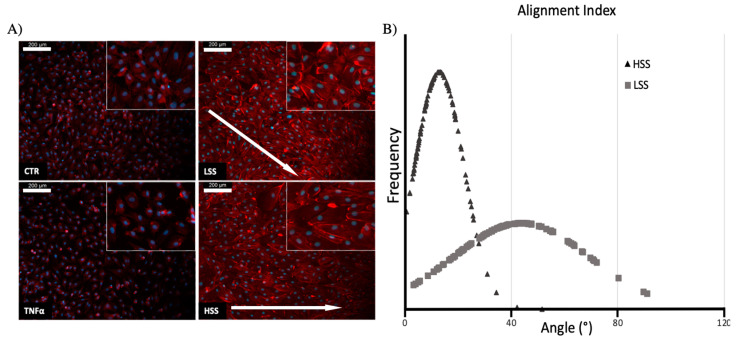
(**A**) F-actin fluorescence analysis on HUVECs cultured in static and dynamic conditions. Nuclei are stained in blue, while actin filaments are stained in red. White arrow indicates the flow direction. (**B**) Quantitative analysis of the angles subtended by the cell’s major axis and the flow direction (*p* < 0.001). Scalebar: 200 μm.

Phalloidin staining was performed to evaluate cell morphology in static and dynamic conditions (Figure 1). Static cultures exhibit the typical cobblestone shape, whereas TNFα-challenged cells are characterized by a slightly reduced cell density without any significant morphological alteration. Dynamic cultured cells are, instead, characterized by the prominent expression of stress fibers (actin) preferentially localized in the periphery of the cell. HUVECs exposed to HSS (10 dyn/cm^2^) show a higher degree of cellular alignment along the direction of the flow. However, a certain degree of cell alignment is also observed in all sheared cells. Images were also analyzed in ImageJ software by calculating the angle subtended between the cell’s major axis and the flow direction.

#### 2.1.2. PECAM-1 (CD31) Expression Depends on Shear Stress Magnitudes

**Figure 2:**
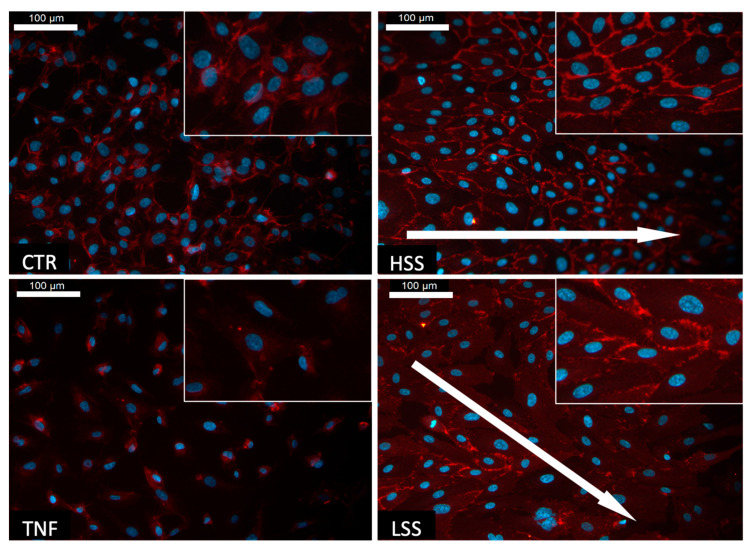
PECAM-1 (CD31) staining in HUVECs cultured in static and dynamic conditions. Represented in blue are the cells’ nuclei, while in red are PECAM-1 proteins. White arrow indicates the flow direction. Scalebar: 100 μm.

The expression of PECAM-1 was evaluated in both static and dynamic cultured samples. The controls show a limited expression of PECAM-1, which significantly decreased when in the presence of TNFα. Samples subjected to shear forces show enhanced expression of PECAM-1 compared to static controls, with a marked peripheral (membrane) localization (Figure 2). Remarkably, regions exposed to HSS display a clear pattern of expression at cell–cell junctions, while regions challenged with LSS are characterized by limited and non-homogeneous expression at junctions.

#### 2.1.3. eNOS and E-selectin Protein Expression Depends on Shear Stress

**Figure 3:**
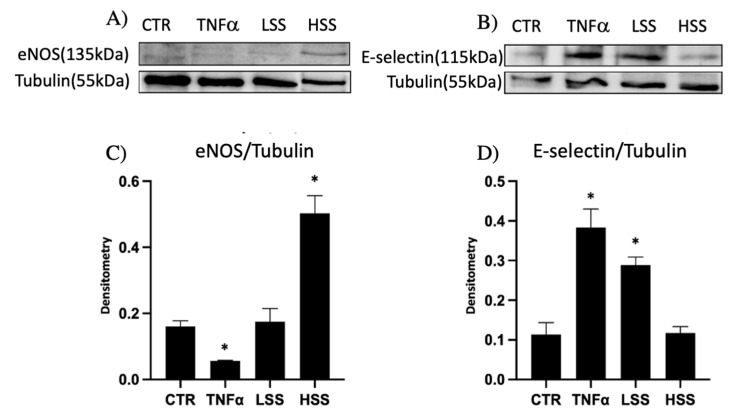
Western blot analysis of eNOS (**A**) and E-selectin (**B**) in HUVECs cultured under shear stress. (**C**,**D**) represent the normalization data of eNOS and E-selectin, respectively (* *p* < 0.05 vs. control).

eNOS and E-selectin protein expression was performed via Western blot given its pivotal role as a marker for endothelial function and dysfunction. eNOS was found to be highly expressed only when HUVECs were subjected to shear stress stimulation at physiological ranges (HSS) (Figure 3A). LSS-challenged endothelial cells, despite showing a higher expression compared to inflammatory control conditions (TNFα), displayed a significant reduction in eNOS expression compared to endothelial cells maintained at HSS. The expression of E-selectin is significantly higher when HUVECs are challenged with pro-inflammatory stimulus TNFα in static conditions or with low shear stress magnitude (LSS), while in static control and HSS samples the expression is lower (Figure 3B). Tubulin expression was used to normalize the results obtained (Figure 3C,D). 

#### 2.1.4. PBMC Adhesion Rate Is Dissimilar in Differently Sheared HUVEC Monolayers

**Figure 4:**
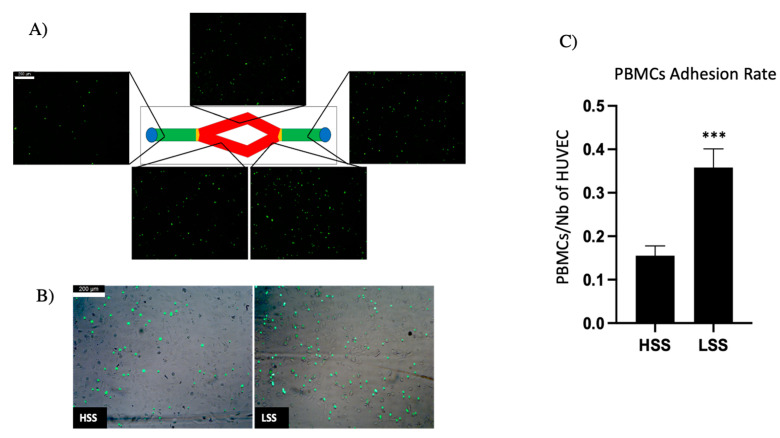
PBMC adhesion assay. (**A**) Leukocyte adhesion on Y-shaped PPFCs; notably, there is a higher adhesion rate in branch segments (indicated in red) compared to straight segments (indicated in green). (**B**) HUVEC monolayer visualized in phase contrast and fluorescent adherent PBMCs. (**C**) Quantification of adhesion rate by fluorescence (*** *p* < 0.005). Scalebar: 200 μm.

Leukocyte adhesion was tested to evaluate the tendency of differently sheared HUVEC monolayers to express adhesion molecules. PBMC adhesion was different depending on the magnitudes of shear stress to which HUVECs were exposed (Figure 4A). More precisely, double-branch regions of Y-shaped PPFCs, which are subjected to LSS, demonstrated a significantly higher tendency to promote PBMC adhesion, whereas regions exposed to physiological ranges of WSS displayed a lower adhesion, comparable to static controls. To quantitatively evaluate the adhesion rate, adherent PBMCs on differently sheared regions were lysed and their fluorescence was evaluated (Figure 4C).

**Figure 5:**
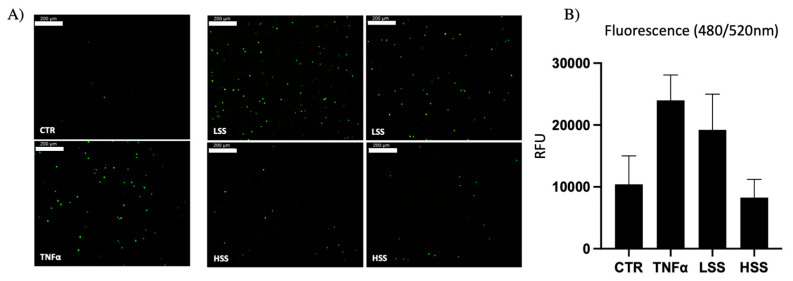
(**A**) PBMC adhesion assay on straight segments of PPFC. (**B**) Quantitative fluorescence analysis. Scalebar: 200 μm.

Moreover, TNFα and LSS samples had the highest PBMC adhesion (Figure 5A,B).

Different PPFC designs only served for the maintenance of the same experimental conditions, mimicking a stenotic event (Y-shaped), or to allow the collection of “single-shear stress” conditioned media (I-shaped).

### 2.2. Conditioned Media Stimulation on HUVEC Cells

To in vitro simulate the paracrine effect of factors secreted by differently sheared endothelial cell monolayers, conditioned media (CMs) from experiments, in which I-Luer PPFCs were used, were collected. In this way, different conditioned media containing the factors released by HUVEC monolayers sheared at low (LSS-CM) or high (HSS-CM) magnitude were collected. Cells were maintained for 24 h with different CMs and subsequently analyzed for different endothelial dysfunction marker and angiogenesis.

#### 2.2.1. HSS-CM and LSS-CM Have Opposite Effects on Cellular Viability

**Figure 6:**
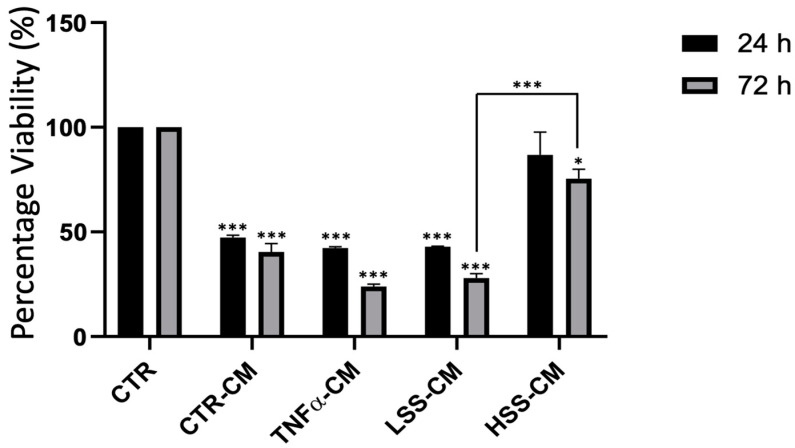
MTT assay on HUVECs subjected to control (CTR), TNFα- and LSS-conditioned medium (CM) for 24 and 72 h. (* *p* < 0.05; *** *p* < 0.005).

Viability assay revealed that HUVEC-conditioned media (HUVEC-CM) allow cell survival over a period of at least 72 h (Figure 6). Interestingly, we found that CTR, TNFα and LSS-CM determined a statistically significant decrease in cell viability at 24 and 72 h (*p* < 0.005) compared to fresh F12K media (CTR). Conversely, HSS-CM determines a higher cell viability compared to CTR-CM maintained up to 72 h. HSS-CM viability was similar to that exhibited by cells cultured in fresh F12K media at 24 h, while a slight reduction was observed at 72 h. This suggests that CM enriched with factors released by endothelial cells sheared at physiological magnitude promotes cell viability. By comparing LSS and HSS-CM samples at 72 h, we found a strong, statistically significant difference (*p* < 0.05).

#### 2.2.2. eNOS and E-selectin Protein Expression in Different CMs Culture

**Figure 7:**
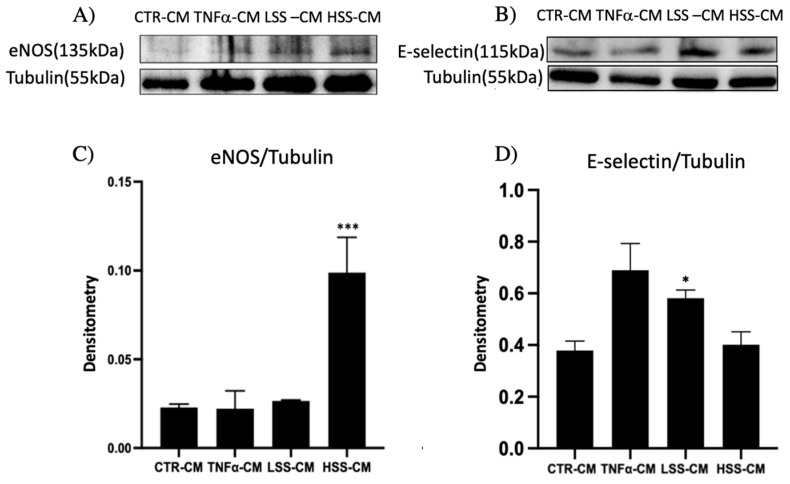
Western blot analysis of HUVEC cell lysates after 24 h of stimulation with TNFα-, LSS- and HSS-conditioned media (-CMs). (**A**,**B**) show the expression of eNOS and E-selectin, respectively, while (**C**,**D**) represent the normalization data by using tubulin expression level (* *p* < 0.05 and *** *p* < 0.005).

eNOS expression was used to assess endothelial function, and the highest expression was found in HUVEC maintained for 24 h in HSS-CM (Figure 7A). As demonstration of an inflammation state, we instead measured the expression of the adhesion molecule E-selectin, whose upregulation is a hallmark of endothelial dysfunction. As shown in Figure 7B, this was higher in the positive inflammatory control (TNFα-CM) and LSS-CM samples. In particular, LSS-CM demonstrated statistical significance compared to the control (*p* < 0.05). The normalization graph (Figure 7C,D) showed a large statistical significance with respect to the control as well all as to all other samples (*p* < 0.005). 

#### 2.2.3. ICAM-1 mRNA Expression Is Upregulated by LSS-CM

**Figure 8:**
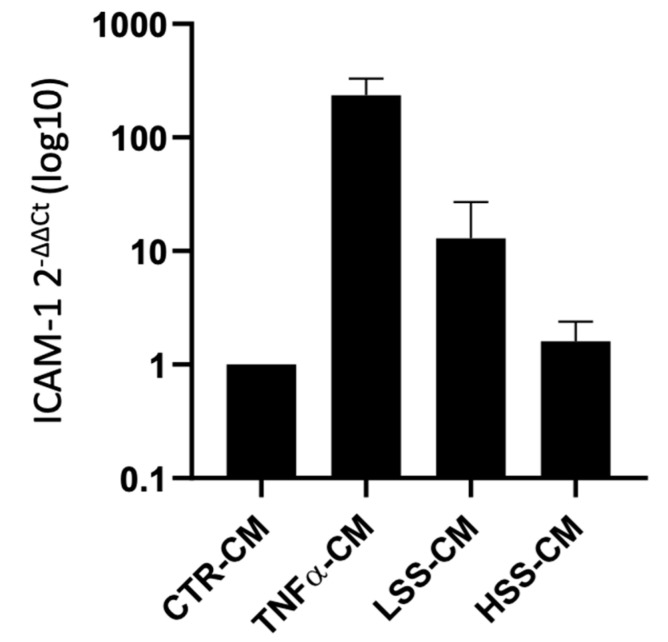
ICAM-1 mRNA expression in HUVECs cultured for 24 h with TNFα-, LSS- and HSS-conditioned media (-CMs). ICAM-1 mRNA expression is represented relative to CTR-CM (2^−ΔΔCt^).

ICAM-1 mRNA expression was evaluated through quantitative real-time RT-PCR. The results of the amplification curves were used to build ΔΔCt graphs (Figure 8). This graph shows the strong induction of ICAM mRNA in TNFα-CM (235-fold). Real-time RT-qPCR data agree with E-selectin protein expression Western blot analysis demonstrating that ICAM-1 mRNA molecules also have a slight increase (13-fold) compared to the static control (CTR-CM). Conversely, HSS-CM is associated with a negligible increase in ICAM-1 mRNA expression (1,6-fold) with respect to the control, suggesting that HUVECs sheared at physiological ranges have limited or no secretion of factors involved in ICAM-1 upregulation. On the other hand, the slight upregulation of ICAM-1 observed on HUVECs cultured in LSS-CM suggests the putative presence of these proinflammatory factors. However, the high variability between different experiments and hence in CMs batches resulted in large standard deviation in ΔΔCt; therefore, statistical significance was not achieved.

#### 2.2.4. PBMC Adhesion Rate Is Higher in LSS-CM-Cultured HUVECs

**Figure 9:**
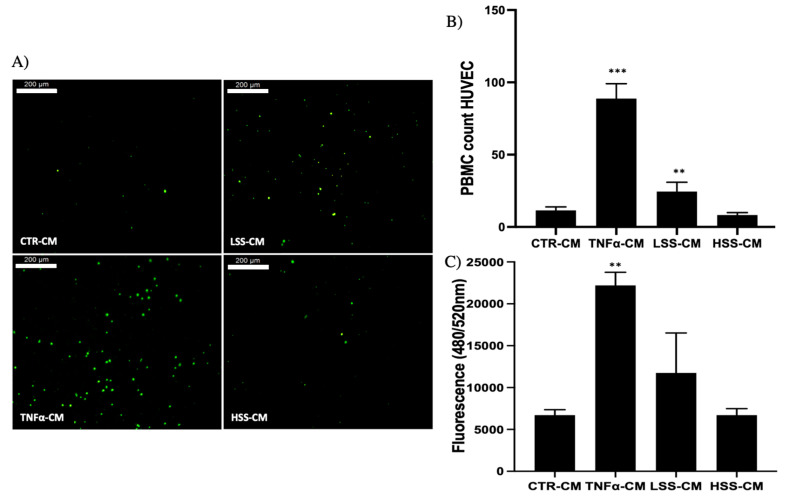
PBMC adhesion assay on HUVEC monolayer cultured for 24 h with TNFα-, LSS- and HSS-conditioned media (-CMs). (**A**) Immunofluorescence imaging of representative fields of the different conditions. (**B**) Adherent PBMC absolute count on HUVEC monolayers. (**C**) Fluorescence quantification of the adherent PBMCs (** *p* < 0.01 and *** *p* < 0.005). Scalebar: 200 μm.

According to previous results showing enhanced ICAM-1 mRNA and E-selectin protein expression, as demonstrated in Figure 9, PBMC adhesion was higher on TNFα- and LSS-CM-challenged endothelial monolayers. PBMC count revealed a strong statistical significance for TNFα-CM and LSS-CM with a *p* < 0.005 and *p* < 0.01, respectively (Figure 9B). Samples were lysed and used to measure the relative fluorescence, which is directly proportional to the number of adherent PBMCs. Once again, these results are in accordance with previous observations, demonstrating the highest adhesion on TNFα-CM and LSS-CM-cultured HUVECs (Figure 9C).

#### 2.2.5. LSS-CM Promotes Tube Formation

**Figure 10:**
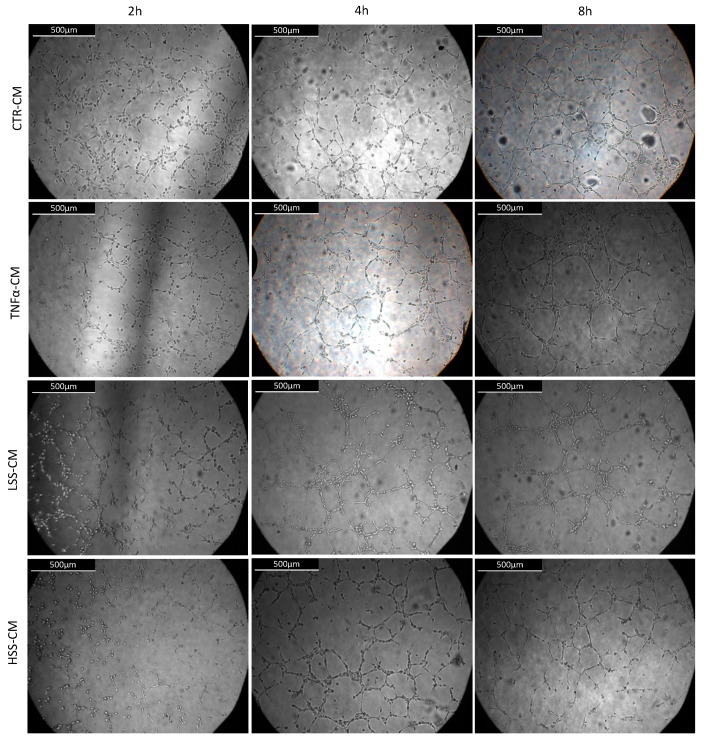
Tube formation assay on Matrigel. Representative bright field images of the same central area were taken at different time points per each condition. Scalebar: 500 μm.

**Figure 11:**
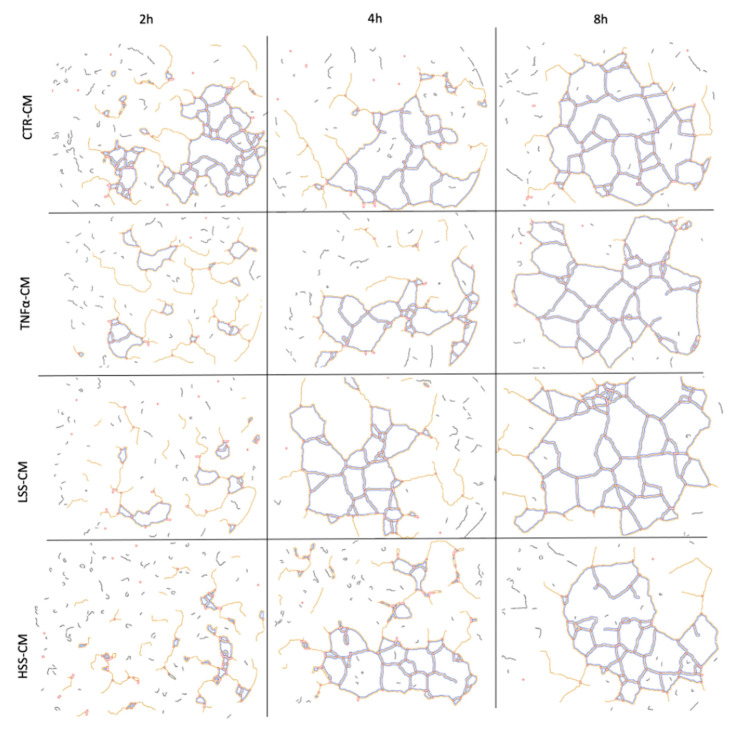
Computer reconstruction obtained in ImageJ Angiogenesis Analyzer plug-in of master segments and junctions observed in HUVECs seeded on Matrigel and stimulated for 8 h with different CMs.

**Figure 12:**
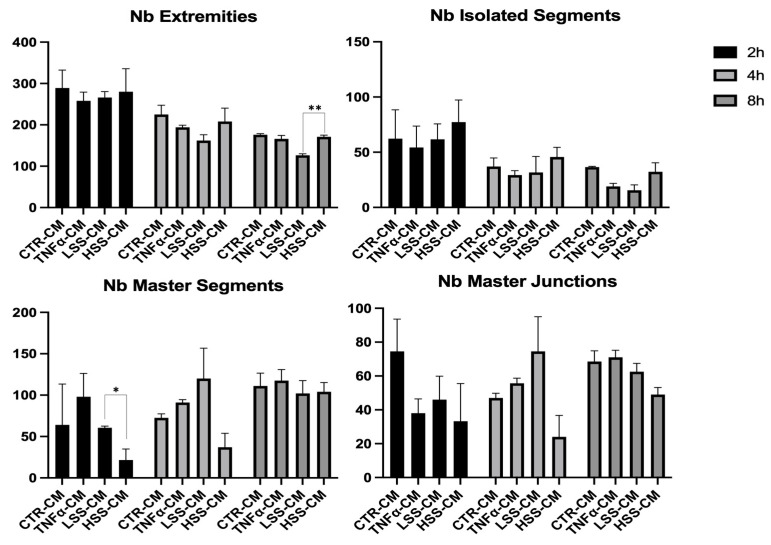
Angiogenesis analysis of tube formation assay at different time points. (* *p* < 0.05, ** *p* < 0.01).

**Figure 13:**
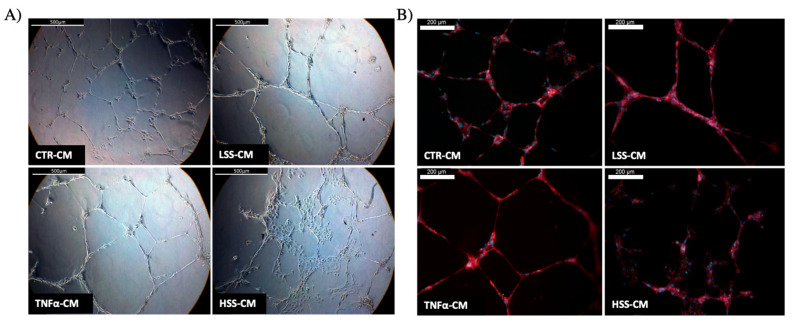
(**A**) Phase contrast (scalebar: 500 μm) and (**B**) tubulin fluorescence images (scalebar: 200 μm) of tube formation at 24 h performed with HUVECs in presence of different conditioned media.

**Figure 14:**
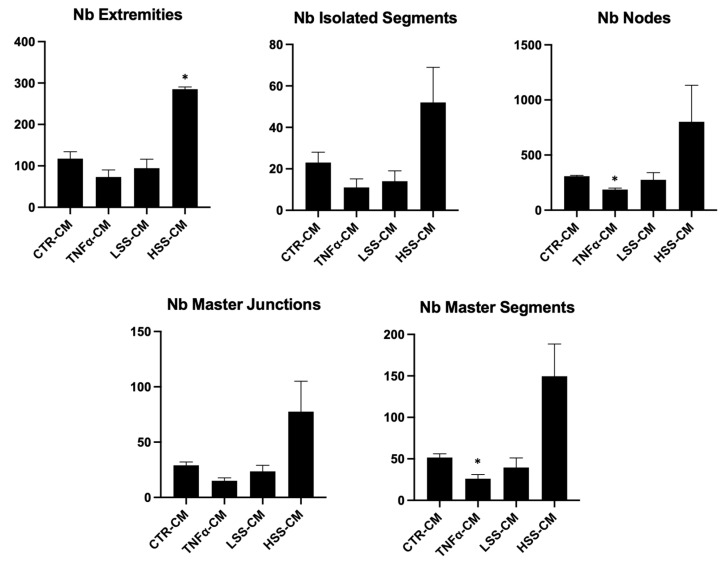
Angiogenesis analysis of tube formation images at 24 h. (* *p* < 0.05).

We then analyzed the angiogenetic effect of the different conditioned medium by evaluating HUVEC tube formation, rendered in Angiogenesis Analyzer software. Although with low or no statistical significance, a tendency to better form tubes in TNFα and LSS-CM is evident (Figure 10 and Figure 11). At 4 and 8 h, a reduction in the number of extremities and isolated segments in HUVEC stimulated with TNFα-CM and LSS-CM is shown (Figure 12). Furthermore, the number of master segments and master junctions is higher in these samples, especially at 4 h. The number of extremities between LSS and HSS-CM is significantly different at 8 h, while the number of master segments is significantly higher at 2 h in LSS-CM compared to HSS-CM. Figure 13 shows the tube structures after 24 h, respectively, in phase contrast and FITC-Phalloidin immunofluorescence. In Figure 14, images taken at 24 h were analyzed by means of Angiogenesis Analyzer software according to Carpentier guidelines [43]. The analysis evidenced the difference between the samples cultured in HSS-CM for which we have higher counts of all parameters compared to other conditions. 

#### 2.2.6. MMP-2 Expression Is Higher in LSS-CM-Cultured HUVECs

**Figure 15:**
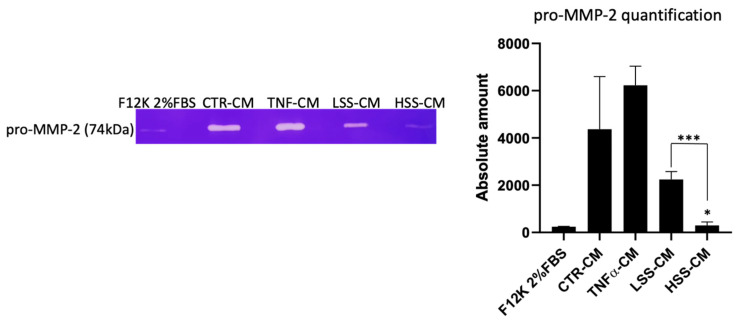
pro-MMP-2 expression evaluated by zymography. On the right, quantitative analysis of the absolute expression of pro-MMP-2 in different conditioned media (CMs) is shown. (* *p* < 0.05, *** *p* < 0.005).

Zymography allowed us to quantitatively compare the type and the amount of metalloproteases released by HUVECs during 24 h WSS stimulation, therefore allowing us to better understand the vascular remodeling potential of different CMs. As shown in Figure 15, pro-MMP-2 is the only metalloproteases found in the conditioned media, while pro-MMP-9 is not observed. The results demonstrate that CMs retrieved from static experiments have higher concentrations of MMP-2 compared to CM from experiments in which HUVECs were challenged with shear stress. Interestingly similar quantities of MMP-2 are found in CTR-CM and TNFα-CM, suggesting that this pro-inflammatory cytokine is only partially responsible for MMP-2 upregulation. On the contrary, LSS-CM and HSS-CM are characterized by large differences in the amount of MMP-2 detected: LSS-CM has 8-fold (*p* < 0.001) higher MMP-2 levels compared to HSS-CM, which has a negligible amount compared to what was observed in fresh F12K 2%FBS medium (Figure 15). Overall, a drop in the amount of released MMP-2 is observed when cells are subjected to shear stress, although this is statistically significant only when cells are sheared at physiological magnitude (10 dyn/cm^2^).

## 3. Discussion

In this manuscript, we successfully designed an in vitro microfluidic model for the study of shear-stress-challenged endothelial cells, and the additional evaluation of downstream effects of their secretory profile. For this specific purpose, Y-shaped chambers, in some instances, allow for the in vitro reproduction of a stenotic event in the blood vessel. Thus, a Y-shaped design allowed us to confidently determine that the morphological changes on the endothelial monolayer were determined by shear stress magnitude differences. Irrespective of this, this experimental approach was not useful to understand the effects of the secretome downstream. Thus, the experiments focused on the effects of the secretome were performed on straight I-Luer PPFCs, to guarantee that the secretome was influenced by a specific shear magnitude only. The Y-shaped chambers allowed us to verify the effects of the shear stress only. In fact, the secretome in these experiments was influenced by both the dynamic conditions. Thus, only morphological differences were considered. Apart from the morphological changes, no other significant differences were observed. In fact, in these conditions, the secretome did not affect the cell behavior in the immediate surrounding, which was related to the shear stress only. To better understand the effects of the secretome, the use of the linear chamber was mandatory, in order to collect separately the secretome related to each shear stress applied. Due to the different secretome content, in these conditions, the differences in terms of cell behavior with respect to the inflammatory response involvement were enhanced.

As previously demonstrated [44,45,46], low shear stress associates with endothelial dysfunction resulting in increased expression of adhesion molecules and, most importantly, reduced protein levels of eNOS, which is a primary hallmark of endothelial dysfunction. Morphological analysis revealed that HUVECs can align in the direction of the flow. There are two main hypotheses guiding endothelial alignment: (I) to better resist shear forces, indeed the cytoskeletal reorganization is pivotal to properly enhance endothelial cells stiffness; (II) to improve the endothelial cells’ ability to sense shear forces and transduce accordingly [47,48]. This response is hypothesized to be related not only to receptor-mediated cell mechanosensing but also to biochemical autocrine and paracrine stimuli following the mechanotransduction. The alignment was much more prominent in HSS segments, while in LSS regions cells were less organized along flow’s direction, as confirmed by the quantitative analysis of the angle formed between cellular soma and flow direction. This is again an indication that LSS is associated with poor endothelial performance; in fact, endothelial cell alignment along the direction of laminar fluid flow is widely understood to be a defining morphological feature of vascular homeostasis [49]. 

eNOS was significantly reduced in all experimental conditions, also on static control, except for HUVECs stimulated in a dynamic culture with a WSS equal to 10 dyn/cm^2^, which closely mimics physiological conditions. This underlines the importance of WSS in maintaining proper endothelial function. eNOS is known to be transcriptionally upregulated early after shear stress application [50], and we demonstrated that the upregulation is maintained for a period of at least 24 h. This upregulation occurs downstream of the mechanosome, which is the complex constituted by PECAM-1 (CD31), VEGFR-2 and VE-Cadherin, located at the periphery of the cell constituting the cell–cell junction, also known as adherens junctions (AJs) [6,8]. Not surprisingly, we found that endothelial cells in regions sheared at low magnitudes had a limited expression of PECAM-1 (CD31) at intercellular junctions, suggesting a limited ability to form adherens junctions. These results agree with observations in both in vitro and ex vivo autoptic studies, demonstrating that laminar shear stress at physiological magnitudes is required to maintain endothelial barrier integrity [51,52]. Clinically, low shear stress regions are associated with leaky junctions and enhanced permeability to low-density lipoproteins (LDL) and PBMCs, basic for atherosclerotic plaque formation. 

Furthermore, given the pivotal role of PECAM-1 (CD31) in the mechanosome responsible for shear-stress-mediated transduction, we hypothesized that limited expression of this protein at junctions results in an impaired regulation of eNOS as well as its lower activation [53]. According to this consideration, eNOS levels were found to be lower in static controls as well as in inflammatory static controls (TNFα 100 ng/mL) and in cells challenged with pathological shear stress, corresponding to 6 dyn/cm^2^ (LSS). Reduced bioavailability of NO is considered as one of the most important factors associated with vascular disease [54,55]. It is unclear whether this is a cause or a result of endothelial dysfunction. There are several factors that affect the production of NO and its ability to reach or diffuse to proper cellular targets, as well as the availability of substrates and cofactors [56].

PBMC adhesion assays confirmed that low shear stress stimulation tends to upregulate the expression of molecules involved in leukocyte adhesion. Additionally, we directly demonstrated the overexpression of E-selectin. Coherently with other works [57], E-selectin is more represented in LSS samples (6 dyn/cm^2^) and as expected in the inflammatory controls (TNFα 100 ng/mL). These results, coupled with the decreased ability to form cell–cell junctions and limited endothelial function, demonstrate that our microfluidic system can reproduce the hallmarks of local low shear stress regions observed in vivo [57]. 

Given these results, the collection of conditioned media after a period of 24 h of dynamic stimulation allowed us to consistently evaluate the secretory profile of both functional (HSS) and dysfunctional (LSS) endothelial cells. Conditioned media demonstrated their potential ability to differently modulate several biological processes, ranging from endothelial function to proinflammatory phenotype and angiogenesis. Despite the simplicity and low cost, the generation and usage of conditioned media has had a primary role in biomedical research enabling the study of both paracrine and autocrine effects in cell culture settings [58,59,60,61,62]. Interestingly, HUVECs maintained in HSS-CM showed an increase in cell viability compared to any other CMs observed at 72 h, while LSS-CM displayed a decrease in cell viability at both 24 and 72 h. Moreover, Western blot analysis for eNOS expression in HUVECs cultured with CM showed an increase in eNOS expression in cells exposed for 24 h to HSS-CM. These differences represent an interesting result since we previously demonstrated that eNOS expression was detected only when cells are subjected to shear stress, while in this case, despite the static culture of HUVEC cells, eNOS expression is high and this could be due to the presence of specific factors in conditioned media able to maintain EC functionality. Other researchers found that increased levels of ROS, whose production is enhanced in endothelial dysfunction, upregulates iNOS and eNOS expression in human coronary artery endothelial cells (HCAECs) both in vitro and in vivo. This effect was justified mainly as an effect of the depletion of NO and only to a lesser extent by a direct ROS-dependent upregulation [63,64]. However, in our experiments, we did not find any eNOS upregulation related to TNFα-CM or LSS-CM culture. 

Leukocyte adhesion assay was performed to evaluate the tendency of isolated PBMCs to adhere to HUVEC monolayers stimulated for 24 h with CM. The results were also supported by real-time reverse transcriptase quantitative PCR analysis to study the expression of ICAM-1 mRNA and Western blot analysis to screen the protein levels of E-selectin. 

These experiments revealed that HUVECs upregulate the expression of adhesion molecules at both the mRNA and protein level when subjected to LSS-CM, as seen with pro-inflammatory CM. This is likely to be caused by the release of pro-inflammatory cytokines, triggering the NF-κB pathway and resulting in an increased production of adhesion proteins [65]. In support of this hypothesis, interleukin (IL)-6 and IL-8 are known to be more expressed by ECs sheared at low magnitude [66], and these cytokines are strong stimulators for adhesion molecule expression [67], including E-selectin. Finally, we evaluated the angiogenic potential of the different conditioned media by performing tube formation assay and gelatin zymography. This was done to evaluate the possibility that regions of the endothelium subjected to LSS in vivo can promote angiogenesis and vascular remodeling in downstream microvascular compartments as a response to inadequate environmental conditions. Indeed, this process would modify upstream hemodynamics resulting in further reduction in shear stress and to the expansion of endothelial regions subjected to atherogenic stimuli, as hypothesized by Siasos and colleagues in 2018 [38,42,68]. Data at 2, 4 and 8 h were used to build a tube formation kinetic profile in which, despite not being statistically significant, it is possible to appreciate the tendency of TNFα-CM and LSS-CM to favor angiogenesis. This tendency is particularly evident early in the experiment after only 4 h of CM stimulation. In fact, in these two conditions we observed a reduction in isolated elements count as well as an increase in the number of master segments and junctions. These parameters are commonly considered positive markers for angiogenesis in literature [43]. Most importantly, at 24 h we can appreciate a more evident difference among the conditions. TNFα- and LSS-conditioned media are characterized by long straight tubular structures resembling mature neovessels [69], while CTR-CM is characterized by a higher number of merges, with several uniformly distributed inter-nodular segments. 

On the other hand, HSS-CM displays a significantly different structure with poor tube formation coupled with a higher cellular count. These cells are not aligned but clustered, and their proliferation pattern resembles the typical structures formed by HUVECs cultured in static conditions. These results are in line with other research groups that demonstrated that sheared HUVECs release basic fibroblast growth factor (bFGF) [70], which is known to strongly induce capillary-like formation in vitro together with other factors such as vascular endothelial growth factor (VEGF), platelet-derived growth factor (PDGF), and epidermal growth factor (EGF) [71]. 

Similar to our experimental model, Russo and colleagues [72] demonstrated that rabbit aortic endothelial cells exposed for 4 h to sub-physiological and physiological values of shear stress have a different tendency to form tube-like structures. Moreover, they confirmed that mRNA expression of pro-angiogenic factors such as vascular endothelial growth factor A (VEGF-A), transforming growth factor-beta 1 (TGF-β1) and transforming growth factor-beta 3 (TGF-β3) is higher in EC cells exposed to pathological shear stresses compared to physiological shear stress. Other research groups demonstrated that human retinal microvascular endothelial cells (HRMECs) challenged with low shear stress magnitude have increased levels of PDGF-B [66] together with inflammatory cytokines and chemokines. This seems to confirm our results, since PDGF is a strong proangiogenic factor [71]. Together with pro-angiogenic factors, metalloproteases (MMPs) are pivotal for new vessels’ growth and vascular remodeling. Metalloproteases type 2 (MMP-2) are secreted in their inactive form (pro-MMP-2) and are further activated by specific receptors such as membrane type 1 MMP (MT1-MMP). They are Gelatinase-A, Type IV Collagenase metalloproteases and hence they play a major role in angiogenesis and vascular remodeling [73,74]. Gelatin zymography demonstrated that HUVECs release different amounts of pro-MMP-2 when subjected to different shear stress magnitudes. In particular, lower levels of MMP-2 were detected in HSS-CM while crescent levels were found in LSS-CM and static controls. Interestingly, the release of MMP-2 in the conditioned media is similar in static controls while it is extremely different in LSS-CM and HSS-CM. In support of our finding, previous studies demonstrated that sub-physiological shear stress promotes the formation of podosomes, which are positively correlated with the metalloprotease release-mediating degradation of the extracellular matrix at the basal surface of cells [75]. Thus, these data suggest that endothelial cells subjected to LSS could be responsible for the release of factors involved in angiogenesis and vascular remodeling, possibly opening the path for interesting insights into hemodynamic correlations between epicardial endothelial dysfunction and microvascular endothelial dysfunction.

These results allowed us to speculate over the possibility that dysfunctional endothelial cells can modulate the cellular biology of downstream endothelial cells, e.g., microvascular endothelial cells. We found that factors released by dysfunctional endothelial cells upregulate the expression of adhesion molecules, promoting PBMC adhesion, and they stimulate tube formation. Additionally, in data not shown in this paper, we found that immortalized Human Microvascular Endothelial Cells 1 (HMEC-1) are characterized by a similar, but slighter, behavior to conditioned media as observed in HUVEC cells. Indeed, HMEC-1 revealed to express higher levels of eNOS when cultured in HSS-CM, while LSS-CM-cultured cells displayed higher PBMC adhesion, justified by the enhanced expression of adhesion molecules, and higher ability to form capillary-like structures in tube formation assay experiments. 

From a translational point of view, these results can integrate with recent studies aimed to clarify the correlation between epicardial and microvascular dysfunction. A significant number of patients undergoing coronarography, due to suspected CAD, are found to have normal-appearing coronary arteries. Epidemiological studies have reported that up to 40% of patients undergoing coronary angiography fall into this category [76], [77]. Moreover, paradoxically increased lumen size may occur during the progression of coronary atherosclerosis due to positive arterial remodeling [78]. In 1986, Cannon and Epstein [79] first introduced the term “microvascular angina” for this patient population since they appeared to be characterized by reduced microvascular dilator capacity. They proposed that dysfunction of small intramural pre-arteriolar coronary arteries might be the pathogenic cause of this syndrome [80,81]. In 2007, Camici and Crea [82] reviewed this subject and proposed a pathogenetic classifications of coronary microvascular dysfunction (CMD). They hypothesized that microvascular angina is a pivotal hallmark in CMD, especially in those patients characterized by the absence of myocardial disease or obstructive atherosclerosis [81]. CMD shares most of the risk factors associated with coronary atherosclerosis. Typically, inflammatory and oxidative stress are considered the most important leading causes, tightly bound to risk factors such as obesity, early menopause, smoking, hypertension, dyslipidemia and insulin-resistance [44,83,84,85,86,87]. Recently, several researchers tried to evaluate the causal relationship between epicardial endothelial dysfunction and microvascular endothelial dysfunction. In 2018, Siasos and colleagues [38] demonstrated that microvascular dysfunction is strictly correlated with epicardial coronary dysfunction but, up to date, no indications about possible biological mechanisms have been proposed. Thus, the contribution of factors released by endothelium subjected to low shear forces to CMD could be related not only to the peripheral induction of endothelial dysfunction, but also to vascular remodeling. This, indeed, could drive deep changes in microvascular hemodynamics that could ultimately affect blood flow in epicardial coronaries, it being a possible reason as to why CMD predisposes an individual to the development of focal atheroma in upstream coronary vessels. 

## 4. Materials and Methods

### 4.1. Cell Culture

Primary Human Umbilical Vein Endothelial Cells HUVEC C-12203 (Sigma Aldrich, Milano, Italy) were used for both dynamic and static experiments. HUVECs were cultured in 75 cm^2^ flasks (Falcon Corning, Berlin, Germany) at 37 °C and 80% relative humidity, in Kaighn’s Modification of Ham’s F-12 Medium (F12K) (ATCC^®^ 30-2004, Manassas, VA, USA) supplemented with Penicillin-Streptomycin-Amphotericin B Solution (Final concentration Penicillin: 0.02 Units/mL, Streptomycin: 0.02 μg/mL, Amphotericin B: 0.05 ng/mL) (Euroclone, Nordhausen, Germany), Endothelial Cell Growth Supplement, EGCS 0.05 mg/mL (Corning, Berlin, Germany) and Heparin 100 μg/mL (Sigma Aldrich, Milano, Italy) with 10% Fetal Bovine Serum (Gibco, Milano, Italy) prior to experiments. During the experimental process HUVECs were cultured with F12K 2% FBS to reduce foaming of the medium in the dynamic flow and to enhance the effect of the dynamic stimulation. Only cells from passage 3 to 6 were used. 

### 4.2. Microfluidic Culture System

HUVECs were subjected for 24 h to different shear stress magnitudes by means of parallel plate flow chambers (PPFCs) purchased from Ibidi (Ibidi GmBH, Gräfelfing, Germany). In particular, µ-Slide I Luer were used to culture HUVECs at sub-physiological 6 dyn/cm^2^ (low shear stress—LSS) and physiological 10 dyn/cm^2^ (high shear stress—HSS) uniform magnitude of shear stress, while µ-Slide Y-shaped (ibidi GmBH, Gräfelfing, Germany) were used to culture HUVECs at different WSS magnitudes, within the same PPFC, depending on the geometry of the plate (<6 dyn/cm^2^ in double-branch regions and 10 dyn/cm^2^ in straight segments). For experiments concerning cell morphology and PBMC adhesion, only Y-shaped chambers were used, in order to experimentally maintain the same conditions (i.e., cell seeding, confluency, environment) with the exception of the shear magnitude, which is not homogeneous on the Y-shaped PPFC, as described, and to reproduce with a good approximation what may occur as a consequence of a stenotic event. Secretome effects were studied and linear chambers were used. Moreover, in order to concentrate the secretome, a smaller amount of medium was used when linear chamber was used.

Shear stress values were calculated as described in literature and according to manufacturer manual.

Briefly, PPFCs allow one to approximate shear stress from a tri-dimensional phenomenon to a bi-dimensional phenomenon, therefore allowing one to culture cells on a slide-like structure. In PPFCs, wall shear stress can be calculated with Equation (2), where *Q* is the flow rate in ml/min, *ƞ* is the medium viscosity in dyne, and *w* and *h* are width and height, respectively, of the rectangular channel.

(2) $$\tau =\frac{6Q\eta }{wh^{2}}$$

The microfluidic system was powered by Masterflex L/S peristaltic pump (Cole Parmer 7550-50, Vernon Hills, IL, USA) and shear stress was adjusted by changing the flow rate upon proper calibration based on the tubing characteristics. Two days before the dynamic stimulation, all the fluidic system setup was autoclaved and placed overnight in the incubator to allow for gas equilibration. According to manufacturer instructions, 100 µL of type I collagen solution (250 µg/mL) was pipetted into the channel as coating for 1 h, then rinsed in PBS. Following this, 100 µL of a 1.5 × 10^6^ cells/mL suspension was plated into the PPFCs channel and allowed to adhere for at least 24 h and to reach >80% confluency. To adequately enrich the medium, the reservoir was filled with 4 mL of F12K 2%FBS medium. After 24 h of shear stress exposure, the PPFCs were disconnected and the media from the reservoir was collected in a 15 mL centrifuge tube, centrifuged at 900 rpm for 5′ to eliminate cellular debris and then stored at −20 °C. Controls were cultured for 24 h in the same medium, with equal surface area/medium volume ratio, but in static conditions. Positive controls for inflammatory markers were cultured in the same medium supplemented with 100 ng/mL TNF-alpha (Cell BioLab Inc., Bergamo, Italy).

### 4.3. Morphological Analysis

After 24 h of stimulation in Y-shaped PPFC and static CTR/TNFα (100 ng/mL), cells were fixed with 4% formalin (Sigma Aldrich, Milano, Italy) for 30′ at room temperature. Phalloidin-Tetramethylrodamin B-Isotiocianate (Tritc) (Sigma Aldrich, Milano, Italy) was incubated over cell monolayer and placed in the incubator for 45′. Nuclei were stained with 4’,6-diamidino-2-phenylindole (DAPI) 1 µg/mL (Sigma Aldrich, Milano, Italy). Stained cells were observed using a fluorescence microscope (Leica DM700), and images were acquired via Leica software LAS V4.7 (Leica, Munich, Germany).

### 4.4. Western Blot

After 24 h of LSS or HSS stimulation, PPFCs were disconnected from the circuit and the media were washed with phosphate-buffered saline (PBS). PPFCs were placed in an ice bath and the cell monolayer was lysed with 50 µL of complete radioimmunoprecipitation assay buffer (RIPA) supplemented with 1:100 PMSF, anti-proteases cocktail (VWR, Milano, Italy) and Na_3_VO_4_ (Sigma Aldrich, Milano, Italy). Samples were centrifuged at 13,000 rpm for 20′ at 4 °C to remove cellular debris and then stored at −20 °C. Experiments on static HUVEC cultured in conditioned media were performed on 6-well plates. HUVEC were seeded at an initial density of 20,000 cells/cm^2^ and allowed to grow until sub-confluency in F12K 10%FBS medium. When confluency was reached (>80%), medium was removed and 1 mL of differently conditioned media was added. After 24 h of conditioned media culture, cell monolayer was rinsed with PBS and lysed as described before. Samples were run on 10% Acrylamide:N, N’ Methylenebisacrylamide 37.5:1 (Sigma Aldrich, Italy) SDS-Page gel and, then, blotted on nitrocellulose membrane (GE HealthCare LifeSciences, Freiburg, Germany). The membrane was then saturated with 5% fat-free milk protein powder in PBS, washed with PBS Tween 0,1% and incubated with anti-eNOS primary antibody 1:500 (MerckMillipore—SAB4502014, Darmstadt, Germany), anti-E-selectin 1:500 (H-300 SantaCruz Biotechnologies, Dallas, TX, USA), anti-alpha-tubulin 1:1000 (MerckMillipore 05-829, Darmstadt, Germany) overnight, that was revealed by HRP-conjugated secondary antibody (SantaCruz Biotechnologies, Dallas, TX, USA). Western blot images were acquired at ChemiDoc (BioRad, Hercules, CA, USA) and further analyzed using ImageJ (NIH, Bethesda, Rockville, MD, USA) and ImageLab (BioRad, Hercules, CA, USA) softwares.

### 4.5. Immunofluorescence Anti-CD31

HUVECs were seeded on Y-shaped PPFCs, while static controls were seeded on 24-well plates and allowed to reach optimal confluency. After 24 h of shear stress stimulation, cell monolayer was fixed with 4% formalin, permeabilized with PBS 1X 0.1% TritonX100 (Sigma Aldrich, Milano, Italy) and saturated with 5% Goat Serum (EuroClone, Milano, Italy) in PBS 1X. Biotinylated antibody anti-CD31 (Bioscience, ThermoFisher Scientific, Clone:390, Munich, Germany) was prepared at 1:50 concentration in 2% Goat Serum (Euroclone, Milano, Italy) and 0,1%TritonX (Sigma Aldrich, Milano, Italy) in PBS1X and incubated overnight at 4 °C. CD31 was revealed by allophycocyanin (APC)-conjugated streptavidin (ThermoFisher Scientific, Munich, Germany) 1:500 in PBS. Nuclei were stained with DAPI 1 µg/mL. Stained cells were observed using a fluorescence microscope (Leica DM700) and images were acquired via Leica software.

### 4.6. Quantitative Real-Time RT-PCR

RT-qPCR was performed on static cultured HUVEC at 80% confluency. After 24 h of different conditioned media (CMs) culture, cell monolayers were rinsed with PBS and lysed in 500 µL of TRIzol (Thermo Fisher Scientific, Milano, Italy) for 5′, and then scraped. Samples were collected in 2 ml tubes and stored at −80 °C until RNA purification, performed with 100 µL of chloroform (Sigma Aldrich, Milano, Italy) and 12,000 rpm centrifugation for 15′ at 4 °C according to the manufacturer’s protocol. The aqueous phase containing RNA was transferred to a clean tube and 250 µL of isopropanol (Sigma Aldrich, Milano, Italy) was added. After 12,000× *g* centrifuge for 15′ at 4 °C, RNA pelleted at the bottom and isopropanol was discarded and replaced with 500 µL of ethanol 75% (Sigma Aldrich, Milano, Italy), spectrophotometrically quantified with Nanodrop (Thermo Fisher Scientific, Milano, Italy) by measuring the optical density at 260 and 280 nm and stored until retro-transcription. Retro-transcription was performed according to High-Capacity RNA-to-cDNA™ Kit (Applied Biosystems, Waltham, MA, USA) manufacturer instructions. cDNA obtained was stored at −20 °C until further use. Real-time quantitative PCR was performed in 10 µL volumes, 9 µL of PCR mix and 1 µL of sample. PCR mix was prepared according to SsoAdvanced Universal SYBR Green Supermix (BioRad, Hercules, CA, USA) protocol with 0.1 mM forward and reverse primers (ICAM-1: for 5′- AGCGGCTGACGTGTGCAGTAAT- 3′ and rev 5′-TCTGAGACCTCTGGCTTCGTCA-3′; GADPH: for 5′-AACGTGTCAGTGGTGGACCTG-3′ and rev 5′- AGTGGGTGTCGCTGTTGAAGT-3′). Strips were placed in CFX Connect Thermal Cycler (BioRad, Hercules, CA, USA), and after amplification completion, the results were exported in CFX Maestro Software (BioRad, Hercules, CA, USA) and analyzed in Excel (Microsoft, Redmont, WA, USA).

### 4.7. Leukocyte Adhesion Assay 

Dynamically cultured HUVECs were stimulated for 24 h at different wall shear stress levels, as described before. Static HUVECs were cultured on 24-well plates, seeded at initial density of 20,000 cell/cm^2^, maintained in standard F12K 10%FBS and allowed to reach confluency. At this point, standard media were discarded and replaced with different conditioned media. PBMCs were isolated from healthy donor using Histopaque^®^-1077 (Sigma Aldrich, Milano, Italy), and cells were counted and aliquoted in cryovials each containing 10^6^ cells/mL in complement-inactivated FBS 10% DMSO (Sigma Aldrich, Milano, Italy), stored at −80 °C for 2 days and later placed in liquid nitrogen until use. On experiment days, cryovials were thawed and washed with serum-free BioWhittakerTM Roswell Park Memorial Institute (RPMI) 1640 (Lonza, Basel, Switzerland). PBMCs were then stained with Leukotracker solution 500X (Cell BioLab Inc., San Diego, CA, USA) properly diluted (1:500), according to manufacturer instructions, for 60′ at 37 °C. Leukotracked PBMCs were then washed and re-suspended in 1 mL of RPMI, 100 µL was pipetted into PPFC channel while 90 µL of the same suspension was plated over static cell monolayers and placed in the incubator for additional 60′ to allow for PBMC adhesion on activated endothelial cells. Cell monolayer was then gently washed with PBS twice and observed using a fluorescence microscope. Images at confluency fields were taken via Leica Microscope Software, 3–5 fields were counted and analyzed. Afterwards, 100 µL RIPA buffer was used to lyse leukotracked PBMC, fluorescence 480/520 nm was read using a Victor X4 Multilabel Plate Reader (Perkin Elmer, Milano, Italy) and data were analyzed in Excel (Microsoft, Redmont, WA, USA).

### 4.8. MTT Assay

Conditioned media collected from sub-physiological LSS, physiological HSS and static CTR/TNFα were used to culture HUVECs over a period of 72 h. Experiments were performed in triplicate, in 96-well plate at a starting cell density of 10,000 cells/cm^2^. A total of 200 µL of conditioned media was used per well. At different time points, conditioned media were discarded and F12K 2% FBS supplemented with 0.5 mg/mL Thiazolyl Blue Tetrazolium Bromide (MTT) (Sigma Aldrich, Milano, Italy) was added. After 4 h, media were discarded and 100 µL of DMSO (Sigma Aldrich, Milano, Italy) was added, allowing for MTT solubilization. Then, 570 nm absorbance was read using Victor4X and data were analyzed in Excel (Microsoft, Redmont, WA, USA).

### 4.9. Tube Formation Assay

Tube Formation assay (TFA) was performed to study the pro-angiogenic potential of conditioned media. In total, 100 µL of Matrigel (Corning, NY, USA) was plated on 24-well plates and allowed to gel at 37 °C in the incubator for 15′. After gelation, cell suspension of HUVECs was prepared and 10,000 cells/well were added at the top of the gel with different CMs and placed in the incubator. Images were taken using a phase contrast microscope every 2 h up to 8 h. After 8 h, the plate was left in the incubator, fixed with formalin 4% at 24 h and stained with Tritc-Phallodin. At least 3 representative images were analyzed in the Angiogenesis Analyzer ImageJ plugin (NIH) according to Carpentier guidelines [43]. Data obtained were collected in Excel (Microsoft, Redmont, WA, USA).

### 4.10. Gelatin Zymography

CMs were quantified via BCA assay and 35 µg of total proteins was run into 0,02% gelatin (Sigma Aldrich, Milano, Italy) acrylamide (Sigma Aldrich, Milano, Italy) SDS-Page gel. After the separation was completed, the gel was washed twice, 90 min each, in 2.5%-Triton (Sigma Aldrich, Milano, Italy) solution, and then incubated with the enzyme-activating solution (10 mM CaCl2, 150 mM NaCl, Trizma base 50 mM at pH 7,4) overnight. The gel was then fixed in 50% methanol and 10% acetic acid solution for 3 h and then stained with Coomassie blue solution (0.05%). After 20 min, the gel was destained with 100 mL of destaining solution (5% methanol and 7% acetic acid). The results were visualized via a transilluminator, and pictures were taken and further analyzed in ImageJ and quantified in Excel (Microsoft, Redmont, WA, USA).

### 4.11. Statistical Analysis

All experiments were repeated in triplicate. All quantitative measurements are represented as means ± standard deviation (SD). Statistical analysis was performed in Excel by means of Student’s t statistical test for homoscedastic samples (=TestT(matrix1;matrix2;two-tails;homoscedastic sample)). The considered statistical significance was α = 0.05. All samples were analyzed with respect to the control, unless otherwise specified. *p*-values are expressed as * *p* < 0.05, ** *p* < 0.01 and *** *p* < 0.005.

## 5. Conclusions

**Figure 16:**
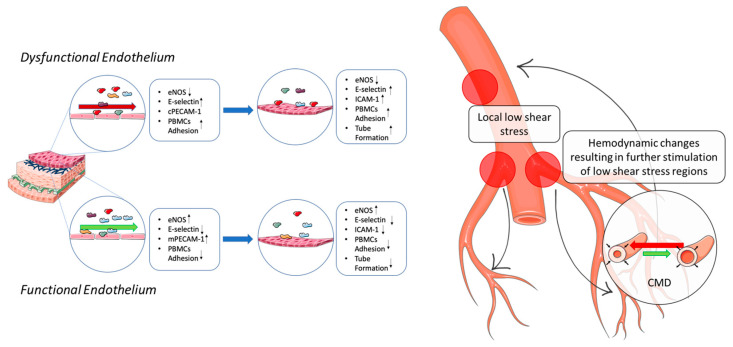
Schematic representation of the results obtained. Endothelial cells (ECs) exposed to LSS are characterized by decreased eNOS levels, an increase in adhesion molecules and the cytoplasmatic localization of PECAM-1 (cPECAM-1), while ECs challenged with HSS display high eNOS level and limited expression of adhesion molecules and PECAM expression at adherens junctions (mPECAM-1). Tube formation is significantly increased in ECs cultured in LSS-CM demonstrating the presence of angiogenic factors in the conditioned media. The image on the right represents a proposed mechanism correlating coronary microvascular dysfunction (CMD) to epicardial coronary artery disease (CAD) associated with low shear stress regions. Coronary arteries are rich in regions experiencing low shear stress during the cardiac cycle, which are often inner curvatures or bifurcations. ECs at these regions are then exposed to low shear forces for a prolonged period before atherogenesis. During this period, it is possible that ECs could release various factors affecting nearby cells as well as downstream microvascular endothelial cells ultimately promoting CMD. In fact, CMD was found to precede in time epicardial CAD, and this is likely to be related to hemodynamic changes in the microvascular compartment affecting upstream blood flow (smart.servier.com).

We demonstrated that dysfunctional endothelial cells could affect downstream endothelium playing a major role in the regulation of adhesion molecule expression and angiogenesis. These in vitro findings suggest that regions physiologically exposed to low shear stress magnitude throughout the coronary epicardial arteries can alter endothelial peripheral (distal) microcirculation, possibly constituting a novel axis for coronary microvascular dysfunction pathogenesis (Figure 16). 

## Abbreviations

CVD	Cardiovascular diseases
IHD	Ischemic heart disease
ECs	Endothelial cells
WSS	Wall shear stress
HUVECs	Human umbelical vein endothelial cells
HSS	High shear stress
HSS-CM	High shear stress conditioned media
LSS	Low shear stress
LSS-CM	Low shear stress conditioned media
eNOS	Endothelial nitric oxide synthase
NO	Nitric oxide
PBMCs	Peripheral blood mononuclear cells
ICAM-1	Intracellular adhesion molecule 1
TNFα	Tumor necrosis factor alpha
TNFα-CM	Tumor necrosis factor alpha conditioned media
PECAM-1	Platelet endothelial cells adhesion molecule 1
VEGFR-2	Vascular endothelial growth factor receptor 2
CAD	Coronary artery disease
E-selectin	Endothelial selectin
AJs	Adherens junctions
LDL	Low density lipoproteins
iNOS	Inducible nitric oxide synthase
ROS	Reactive oxygen species
HCAECs	Human coronary artery endothelial cells
NF-κB	Nuclear factor kappa-light-chain-enhancer of activated B cells
IL-6	Interleukin 6
IL-8	Interleukin 8
VEGF	Vascular endothelial growth factor
PDGF	Platelet derived growth factor
EGF	Epidermal growth factor
TGF	Transforming growth factor
HRMEC	Human retinal microvascular endothelial cells
pro-MMPs	Pro-metalloproteases
MMPs	Metalloproteases
HMEC-1	Human microvascular endothelial cells
CMD	Coronary microvascular disease

## References

[1] Loukas M., Clarke P., Tubbs R.S., Kapos T., Trotz M. (2008). The His family and their contributions to cardiology. Int. J. Cardiol..

[2] Aird W.C. (2015). Endothelium and haemostasis. Hamostaseologie.

[3] Félétou M. (2011). The Endothelium, Part I: Multiple Functions of the Endothelial Cells—Focus on Endothelium-Derived Vasoactive Mediators.

[4] Gordon E., Schimmel L., Frye M. (2020). The Importance of Mechanical Forces for in vitro Endothelial Cell Biology. Front. Physiol..

[5] Roux E., Bougaran P., Dufourcq P., Couffinhal T. (2020). Fluid Shear Stress Sensing by the Endothelial Layer. Front. Physiol..

[6] Tzima E., Irani-Tehrani M., Kiosses W.B., Dejana E., Schultz D.A., Engelhardt B., Cao G., Delisser H.M., Schwartz M.A. (2005). A mechanosensory complex that mediates the endothelial cell response to fluid shear stress. Nature.

[7] Conway D.E., Breckenridge M.T., Hinde E., Gratton E., Chen C., Schwartz M.A. (2013). Fluid Shear Stress on Endothelial Cells Modulates Mechanical Tension across VE-Cadherin and PECAM-1. Curr. Biol..

[8] Coon B.G., Baeyens N., Han J., Budatha M., Ross T.D., Fang J.S., Yun S., Thomas J.-L., Schwartz M.A. (2015). Intramembrane binding of VE-cadherin to VEGFR2 and VEGFR3 assembles the endothelial mechanosensory complex. J. Cell Biol..

[9] Chistiakov D.A., Orekhov A.N., Bobryshev Y.V. (2017). Effects of Shear Stress on Endothelial Cells: Go with the Flow.

[10] Lu T., Barreuther M., Davis S., Madri J.A. (1997). Platelet Endothelial Cell Adhesion Molecule-1 Is Phosphorylatable by c-Src, Binds Src-Src homology 2 Domain, and Exhibits Immunoreceptor Tyrosine-based Activation Motif-like Properties. J. Biol. Chem..

[11] Osawa M., Masuda M., Harada N., Lopes R.B., Fujiwara K. (1997). Tyrosine phosphorylation of platelet enclothelial cell adhesion molecule-1 (PECAM-1, CD31) in mechanically stimulated vascular endothelial cells. Eur. J. Cell Biol..

[12] Xiao Z., Zhang Z., Diamond S.L. (1997). Shear stress induction of the endothelial nitric oxide synthase gene is calcium-dependent but not calcium-activated. J. Cell. Physiol..

[13] Davis M.E., Grumbach I., Fukai T., Cutchins A., Harrison D.G. (2004). Shear Stress Regulates Endothelial Nitric-oxide Synthase Promoter Activity through Nuclear Factor κB Binding. J. Biol. Chem..

[14] Nishida K., Harrison D.G., Navas J.P., A Fisher A., Dockery S.P., Uematsu M., Nerem R.M., Alexander R.W., Murphy T.J. (1992). Molecular cloning and characterization of the constitutive bovine aortic endothelial cell nitric oxide synthase. J. Clin. Investig..

[15] Marsden P., Heng H., Scherer S., Stewart R., Hall A., Shi X., Tsui L., Schappert K. (1993). Structure and chromosomal localization of the human constitutive endothelial nitric oxide synthase gene. J. Biol. Chem..

[16] Chatterjee S. (2018). Endothelial Mechanotransduction, Redox Signaling and the Regulation of Vascular Inflammatory Pathways. Front. Physiol..

[17] Green J., Yurdagul A., McInnis M.C., Albert P., Orr A.W. (2014). Flow patterns regulate hyperglycemia-induced subendothelial matrix remodeling during early atherogenesis. Atherosclerosis.

[18] Zhang J.-X., Qu X.-L., Chu P., Xie D.-J., Zhu L.-L., Chao Y.-L., Li L., Zhang J.-L., Chen S.-L. (2018). Low shear stress induces vascular eNOS uncoupling via autophagy-mediated eNOS phosphorylation. Biochim. Biophys. Acta (BBA)-Mol. Cell Res..

[19] Lin M.I., Fulton D., Babbitt R., Fleming I., Busse R., Pritchard K.A., Sessa W.C. (2003). Phosphorylation of Threonine 497 in Endothelial Nitric-oxide Synthase Coordinates the Coupling of l-Arginine Metabolism to Efficient Nitric Oxide Production. J. Biol. Chem..

[20] Davies P., Remuzzi A., Gordon E.J., Dewey C.F., Gimbrone M.A. (1986). Turbulent fluid shear stress induces vascular endothelial cell turnover in vitro. Proc. Natl. Acad. Sci. USA.

[21] Cunningham K.S., Gotlieb A.I. (2005). The role of shear stress in the pathogenesis of atherosclerosis. Lab. Investig..

[22] Cheng C., Tempel D., Van Haperen R., Van Der Baan A., Grosveld F., Daemen M., Krams R., De Crom R. (2006). Atherosclerotic lesion size and vulnerability are determined by patterns of fluid shear stress. Circulation.

[23] Kwak B.R., Bäck M., Bochaton-Piallat M.-L., Caligiuri G., Daemen M., Davies P., Hoefer I.E., Holvoet P., Jo H., Krams R. (2014). Biomechanical factors in atherosclerosis: Mechanisms and clinical implications. Eur. Hear. J..

[24] Pedrigi R.M., Mehta V.V., Bovens S.M., Mohri Z., Poulsen C.B., Gsell W., Tremoleda J.L., Towhidi L., de Silva R., Petretto E. (2016). Influence of shear stress magnitude and direction on atherosclerotic plaque composition. R. Soc. Open Sci..

[25] Dunn J., Qiu H., Kim S., Jjingo D., Hoffman R., Kim C.W., Jang I., Son D.J., Kim D., Pan C. (2014). Flow-dependent epigenetic DNA methylation regulates endothelial gene expression and atherosclerosis. J. Clin. Investig..

[26] Mitra R., Qiao J., Madhavan S., O’Neil G.L., Ritchie B., Kulkarni P., Sridhar S., Van De Ven A.L., Kemmerling E.M.C., Ferris C. (2018). The comparative effects of high fat diet or disturbed blood flow on glycocalyx integrity and vascular inflammation. Transl. Med. Commun..

[27] Nam D., Ni C.-W., Rezvan A., Suo J., Budzyn K., Llanos A., Harrison D., Giddens D., Jo H. (2009). Partial carotid ligation is a model of acutely induced disturbed flow, leading to rapid endothelial dysfunction and atherosclerosis. Am. J. Physiol.-Heart Circ. Physiol..

[28] Souilhol C., Serbanovic-Canic J., Fragiadaki M., Chico T.J., Ridger V., Roddie H., Evans P.C. (2020). Endothelial Responses to Shear Stress in Atherosclerosis: A Novel Role for Developmental Genes.

[29] Glagov S., Zarins C., Giddens D.P., Ku D.N. (1988). Hemodynamics and atherosclerosis. Insights and perspectives gained from studies of human arteries. Arch. Pathol. Lab. Med..

[30] Carallo C., Tripolino C., De Franceschi M.S., Irace C., Xu X.Y., Gnasso A. (2016). Carotid endothelial shear stress reduction with aging is associated with plaque development in twelve years. Atherosclerosis.

[31] Gnasso A., Irace C., Carallo C., De Franceschi M.S., Motti C., Mattioli P.L., Pujia A. (1997). In vivo association between low wall shear stress and plaque in subjects with asymmetrical carotid atherosclerosis. Stroke.

[32] O’Keeffe L.M., Muir G., Piterina A.V., McGloughlin T. (2009). Vascular Cell Adhesion Molecule-1 Expression in Endothelial Cells Exposed to Physiological Coronary Wall Shear Stresses. J. Biomech. Eng..

[33] Zarins C.K., Giddens D.P., Bharadvaj B.K., Sottiurai V.S., Mabon R.F., Glagov S. (1983). Carotid bifurcation atherosclerosis. Quantitative correlation of plaque localization with flow velocity profiles and wall shear stress. Circ. Res..

[34] Asakura T., Karino T. (1990). Flow patterns and spatial distribution of atherosclerotic lesions in human coronary arteries. Circ. Res..

[35] Friedman M.H., Brinkman A.M., Qin J.J., Seed W. (1993). Relation between coronary artery geometry and the distribution of early sudanophilic lesions. Atherosclerosis.

[36] Ku K.H., Subramaniam N., Marsden P.A. (2019). Epigenetic Determinants of Flow-Mediated Vascular Endothelial Gene Expression. Hypertension.

[37] Chiu J.-J., Chien S. (2011). Effects of Disturbed Flow on Vascular Endothelium: Pathophysiological Basis and Clinical Perspectives. Physiol. Rev..

[38] Siasos G., Tsigkou V., Zaromytidou M., Sara J.D., Varshney A., Coskun A.U., Lerman A., Stone P.H. (2018). Role of Local Coronary Blood Flow Patterns and Shear Stress on the Development of Microvascular and Epicardial Endothelial Dysfunction and Coronary Plaque.

[39] Stone P.H., Saito S., Takahashi S., Makita Y., Nakamura S., Kawasaki T., Takahashi A., Katsuki T., Nakamura S., Namiki A. (2012). Prediction of Progression of Coronary Artery Disease and Clinical Outcomes Using Vascular Profiling of Endothelial Shear Stress and Arterial Plaque Characteristics: The PREDICTION study. Circulation.

[40] Stone P.H., Coskun A.U., Kinlay S., Clark M.E., Sonka M., Wahle A., Ilegbusi O.J., Yeghiazarians Y., Popma J.J., Orav J. (2003). Effect of Endothelial Shear Stress on the Progression of Coronary Artery Disease, Vascular Remodeling, and In-Stent Restenosis in Humans: In vivo 6-month follow-up study. Circulation.

[41] Stone G.W., Maehara A., Lansky A.J., De Bruyne B., Cristea E., Mintz G.S., Mehran R., McPherson J., Farhat N., Marso S.P. (2011). A Prospective Natural-History Study of Coronary Atherosclerosis. N. Engl. J. Med..

[42] Siasos G., Sara J.D., Zaromytidou M., Park K.H., Coskun A.U., Lerman L.O., Oikonomou E., Maynard C.C., Fotiadis D., Stefanou K. (2018). Local Low Shear Stress and Endothelial Dysfunction in Patients With Nonobstructive Coronary Atherosclerosis. J. Am. Coll. Cardiol..

[43] Carpentier G., Berndt S., Ferratge S., Rasband W., Cuendet M., Uzan G., Albanese P. (2020). Angiogenesis Analyzer for ImageJ—A comparative morphometric analysis of “Endothelial Tube Formation Assay” and “Fibrin Bead Assay”. Sci. Rep..

[44] Chatzizisis Y.S., Coskun A.U., Jonas M., Edelman E.R., Feldman C.L., Stone P.H. (2007). Role of Endothelial Shear Stress in the Natural History of Coronary Atherosclerosis and Vascular Remodeling: Molecular, Cellular, and Vascular Behavior. J. Am. Coll. Cardiol..

[45] Zhao Y., Ren P., Li Q., Umar S.A., Yang T., Dong Y., Yu F., Nie Y. (2020). Low Shear Stress Upregulates CX3CR1 Expression by Inducing VCAM-1 via the NF-κB Pathway in Vascular Endothelial Cells. Cell Biophys..

[46] Xie X., Wang F., Zhu L., Yang H., Pan D., Liu Y., Qu X., Gu Y., Li X., Chen S. (2020). Low shear stress induces endothelial cell apoptosis and monocyte adhesion by upregulating PECAM-1 expression. Mol. Med. Rep..

[47] Sathanoori R., Bryl-Gorecka P., Müller C.E., Erb L., Weisman G.A., Olde B., Erlinge D. (2016). P2Y2 receptor modulates shear stress-induced cell alignment and actin stress fibers in human umbilical vein endothelial cells. Cell. Mol. Life Sci..

[48] Levesque M.J., Nerem R.M. (1985). The Elongation and Orientation of Cultured Endothelial Cells in Response to Shear Stress. J. Biomech. Eng..

[49] Steward R., Tambe D., Hardin C.C., Krishnan R., Fredberg J.J. (2015). Fluid shear, intercellular stress, and endothelial cell alignment. Am. J. Physiol.-Cell Physiol..

[50] Davis M.E., Cai H., Drummond G.R., Harrison D.G. (2001). Shear stress regulates endothelial nitric oxide synthase expression through c-Src by divergent signaling pathways. Circ. Res..

[51] Yang F., Zhang Y., Zhu J., Wang J., Jiang Z., Zhao C., Yang Q., Huang Y., Yao W., Pang W. (2020). Laminar Flow Protects Vascular Endothelial Tight Junctions and Barrier Function via Maintaining the Expression of Long Non-coding RNA MALAT1. Front. Bioeng. Biotechnol..

[52] Seebach J., Dieterich P., Luo F., Schillers H., Vestweber D., Oberleithner H., Galla H.-J., Schnittler H.-J. (2000). Endothelial barrier function under laminar fluid shear stress. Lab. Investig..

[53] McCormick M.E., Goel R., Fulton D., Oess S., Newman D., Tzima E. (2011). PECAM-1 regulates eNOS activity and localization through STAT3-dependent NOSTRIN expression. Arterioscler. Thromb. Vasc. Biol..

[54] Förstermann U., Münzel T. (2006). Endothelial Nitric Oxide Synthase in Vascular Disease: From marvel to menace. Circulation.

[55] Förstermann U., Xia N., Li H. (2017). Roles of Vascular Oxidative Stress and Nitric Oxide in the Pathogenesis of Atherosclerosis. Circ. Res..

[56] Chatterjee A., Catravas J.D. (2008). Endothelial nitric oxide (NO) and its pathophysiologic regulation. Vasc. Pharmacol..

[57] Davies P.F., Civelek M., Fang Y., Fleming I. (2013). The atherosusceptible endothelium: Endothelial phenotypes in complex haemodynamic shear stress regions in vivo. Cardiovasc. Res..

[58] Jabbari N., Nawaz M., Rezaie J. (2019). Bystander effects of ionizing radiation: Conditioned media from X-ray irradiated MCF-7 cells increases the angiogenic ability of endothelial cells. Cell Commun. Signal..

[59] Shen C., Lie P., Miao T., Tianyu M., Lu Q., Feng T., Li J., Zu T., Liu X., Li H. (2015). Conditioned medium from umbilical cord mesenchymal stem cells induces migration and angiogenesis. Mol. Med. Rep..

[60] Purushothaman A. (2019). Exosomes from Cell Culture-Conditioned Medium: Isolation by Ultracentrifugation and Characterization. The Extracellular Matrix.

[61] Dai M., Zhang Y., Yu M., Tian W. (2016). Therapeutic applications of conditioned medium from adipose tissue. Cell Prolif..

[62] Bogatcheva N.V., Coleman M.E. (2019). Conditioned Medium of Mesenchymal Stromal Cells: A New Class of Therapeutics.

[63] Zhen J., Lu H., Wang X.Q., Vaziri N.D., Zhou X.J. (2008). Upregulation of Endothelial and Inducible Nitric Oxide Synthase Expression by Reactive Oxygen Species. Am. J. Hypertens..

[64] Ding H., Aljofan M., Triggle C. (2007). Oxidative stress and increased eNOS and NADPH oxidase expression in mouse microvessel endothelial cells. J. Cell. Physiol..

[65] Zhang C. (2008). The role of inflammatory cytokines in endothelial dysfunction. Basic Res. Cardiol..

[66] Ishibazawa A., Nagaoka T., Yokota H., Ono S., Yoshida A. (2013). Low shear stress up-regulation of proinflammatory gene expression in human retinal microvascular endothelial cells. Exp. Eye Res..

[67] Wung B.S., Ni C.W., Wang D.L. (2005). ICAM-1 induction by TNFα and IL-6 is mediated by distinct pathways via Rac in endothelial cells. J. Biomed. Sci..

[68] Gutierrez-Chico J.L. (2018). Endothelial Function and Shear Stress: Which Came First, the Chicken or the Egg?. J. Am. Coll. Cardiol..

[69] Arnaoutova I., George J., Kleinman H.K., Benton G. (2009). The endothelial cell tube formation assay on basement membrane turns 20: State of the science and the art. Angiogenesis.

[70] Gloe T., Sohn H.Y., Meininger G.A., Pohl U. (2002). Shear Stress-induced Release of Basic Fibroblast Growth Factor from Endothelial Cells Is Mediated by Matrix Interaction via Integrin αVβ3. J. Biol. Chem..

[71] DeCicco-Skinner K.L., Henry G., Cataisson C., Tabib T., Gwilliam J.C., Watson N.J., Bullwinkle E.M., Falkenburg L., O’Neill R.C., Morin A. (2014). Endothelial Cell Tube Formation Assay for the In Vitro Study of Angiogenesis. J. Vis. Exp..

[72] Russo T.A., Banuth A.M.M., Nader H.B., Dreyfuss J.L. (2020). Altered shear stress on endothelial cells leads to remodeling of extracellular matrix and induction of angiogenesis. PLoS ONE.

[73] Wang X., Khalil R.A. (2018). Matrix Metalloproteinases, Vascular Remodeling, and Vascular Disease. Adv. Pharmacol..

[74] Rojiani M.V., Alidina J., Esposito N., Rojiani A.M. (2010). Expression of MMP-2 correlates with increased angiogenesis in CNS metastasis of lung carcinoma. Int. J. Clin. Exp. Pathol..

[75] Linder S. (2007). The matrix corroded: Podosomes and invadopodia in extracellular matrix degradation. Trends Cell Biol..

[76] Patel M.R., Peterson E.D., Dai D., Brennan J.M., Redberg R.F., Anderson H.V., Brindis R.G., Douglas P.S. (2010). Low Diagnostic Yield of Elective Coronary Angiography. N. Engl. J. Med..

[77] Jespersen L., Hvelplund A., Abildstrøm S.Z., Pedersen F., Galatius S., Madsen J.K., Jørgensen E., Kelbaek H., Prescott E. (2012). Stable angina pectoris with no obstructive coronary artery disease is associated with increased risks of major adverse cardiovascular events. Eur. Hear. J..

[78] Sipahi I., Tuzcu E.M., Schoenhagen P., Nicholls S.J., Chen M.S., Crowe T., Loyd A.B., Kapadia S., Nissen S.E. (2006). Paradoxical increase in lumen size during progression of coronary atherosclerosis: Observations from the REVERSAL trial. Atherosclerosis.

[79] Epstein S.E., Cannon R.O. (1986). Site of increased resistance to coronary flow in patients with angina pectoris and normal epicardial coronary arteries. J. Am. Coll. Cardiol..

[80] Camici P.G., Rimoldi O.E. (2009). The Clinical Value of Myocardial Blood Flow Measurement. J. Nucl. Med..

[81] Crea F., Camici P.G., Merz C.N.B. (2014). Coronary microvascular dysfunction: An update. Eur. Hear. J..

[82] Camici P.G., Crea F. (2007). Coronary Microvascular Dysfunction. N. Engl. J. Med..

[83] Tarbell J.M. (2010). Shear stress and the endothelial transport barrier. Cardiovasc. Res..

[84] Hwang J., Ing M.H., Salazar A., Lassègue B., Griendling K., Navab M., Sevanian A., Hsiai T.K. (2003). Pulsatile Versus Oscillatory Shear Stress Regulates NADPH Oxidase Subunit Expression: Implication for Native LDL Oxidation. Circ. Res..

[85] Ai L., Rouhanizadeh M., Wu J.C., Takabe W., Yu H., Alavi M., Li R., Chu Y., Miller J., Heistad D.D. (2008). Shear stress influences spatial variations in vascular Mn-SOD expression: Implication for LDL nitration. Am. J. Physiol.-Cell Physiol..

[86] Schoenhagen P., Ziada K.M., Kapadia S.R., Crowe T.D., Nissen S.E., Tuzcu E.M. (2000). Extent and Direction of Arterial Remodeling in Stable Versus Unstable Coronary Syndromes: An intravascular ultrasound study. Circulation.

[87] Hwang J., Rouhanizadeh M., Hamilton R.T., Lin T.C., Eiserich J.P., Hodis H.N., Hsiai T.K. (2006). 17β-Estradiol reverses shear-stress-mediated low density lipoprotein modifications. Free. Radic. Biol. Med..

